# Case report: Hypertrophic lichen planus initially misdiagnosed as squamous cell carcinoma

**DOI:** 10.3389/fmed.2024.1342501

**Published:** 2024-05-15

**Authors:** Nidhi Kuchimanchi, Lydia A. Luu, Preeya T. Shah, Jennifer DeSimone

**Affiliations:** ^1^School of Medicine, University of Virginia, Charlottesville, VA, United States; ^2^Department of Dermatology, University of Virginia, Charlottesville, VA, United States; ^3^Inova Schar Cancer Institute, Fairfax, VA, United States

**Keywords:** hypertrophic lichen planus, cutaneous squamous cell carcinoma, misdiagnosis, topical corticosteroids, oral retinoids, JAK inhibitors, clinicopathological correlation

## Abstract

Fewer than 26 case reports describe hypertrophic lichen planus (HLP) misdiagnosed as cutaneous squamous cell carcinoma (cSCC). It can be difficult to distinguish between HLP and cSCC, as these two conditions share many clinical and histopathological characteristics. Patients who are misdiagnosed with cSCC often undergo unnecessary medical and/or surgical interventions before receiving a diagnosis of HLP and improving on HLP-directed therapy. This case series highlights the course of three female patients, referred to a single tertiary institution between 2018 and 2022, who were initially misdiagnosed with cSCC before receiving a diagnosis of HLP. We have emphasized the clinical and histopathological distinguishing features between HLP and cSCC, the pathogenesis of HLP, and current and new HLP-directed therapy. We hope that this case series serves as a reminder to dermatologists, dermatologic surgeons, and dermatopathologists to be aware of this diagnostic challenge.

## Introduction

Hypertrophic lichen planus (HLP) is a cutaneous subtype of the T-cell-mediated autoimmune disorder, lichen planus, a chronic inflammatory disease that can affect the skin, scalp, oral mucosa, esophagus, and anogenital area (1–3). HLP commonly presents on the lower extremities as pruritic papules and verrucous, polygonal plaques with white lines known as Wickham striae (4, 5). Histologically, HLP presents as hyperplasia limited to the epidermis, lymphocytic infiltration of the dermoepidermal junction, hypergranulosis, acanthosis, and hyperorthokeratosis (6). HLP is also characterized by features of pseudoepitheliomatous hyperplasia, making it difficult to distinguish from cutaneous squamous cell carcinoma (cSCC) (7). The similar clinical and histopathological characteristics between HLP and cSCC often create diagnostic challenges for dermatologic providers. Additionally, HLP can exhibit malignant transformation into cSCC (8–10). This uncertainty, along with the discomfort that is associated with HLP, may result in either aggressive or inadequate treatment strategies that can undermine the patient’s quality of life (5, 10).

Here, we report a case series of three female patients who were referred to a single tertiary center with a diagnosis of cSCC and were ultimately diagnosed with HLP after further review of biopsy results and their entire clinical picture. We suggest that the challenge of diagnosing HLP can be overcome with careful clinicopathological correlation. It is important for clinicians to understand the pathogenesis of HLP and have good clarity regarding diagnoses of “multifocal cSCC,” especially when patients are not responding to therapy. We also discuss treatment options for HLP, including oral retinoids in combination with high-potency topical steroids along with newer therapeutic options including JAK inhibitors (11). We hope to provide dermatologists, dermatologic surgeons, and dermatopathologists with the clinical and histopathologic characteristics of HLP and HLP-directed treatment strategies that are crucial to distinguishing HLP from cSCC to decrease patient morbidity from unnecessary interventions.

## Case report

### Patient 1

**Figure 1:**
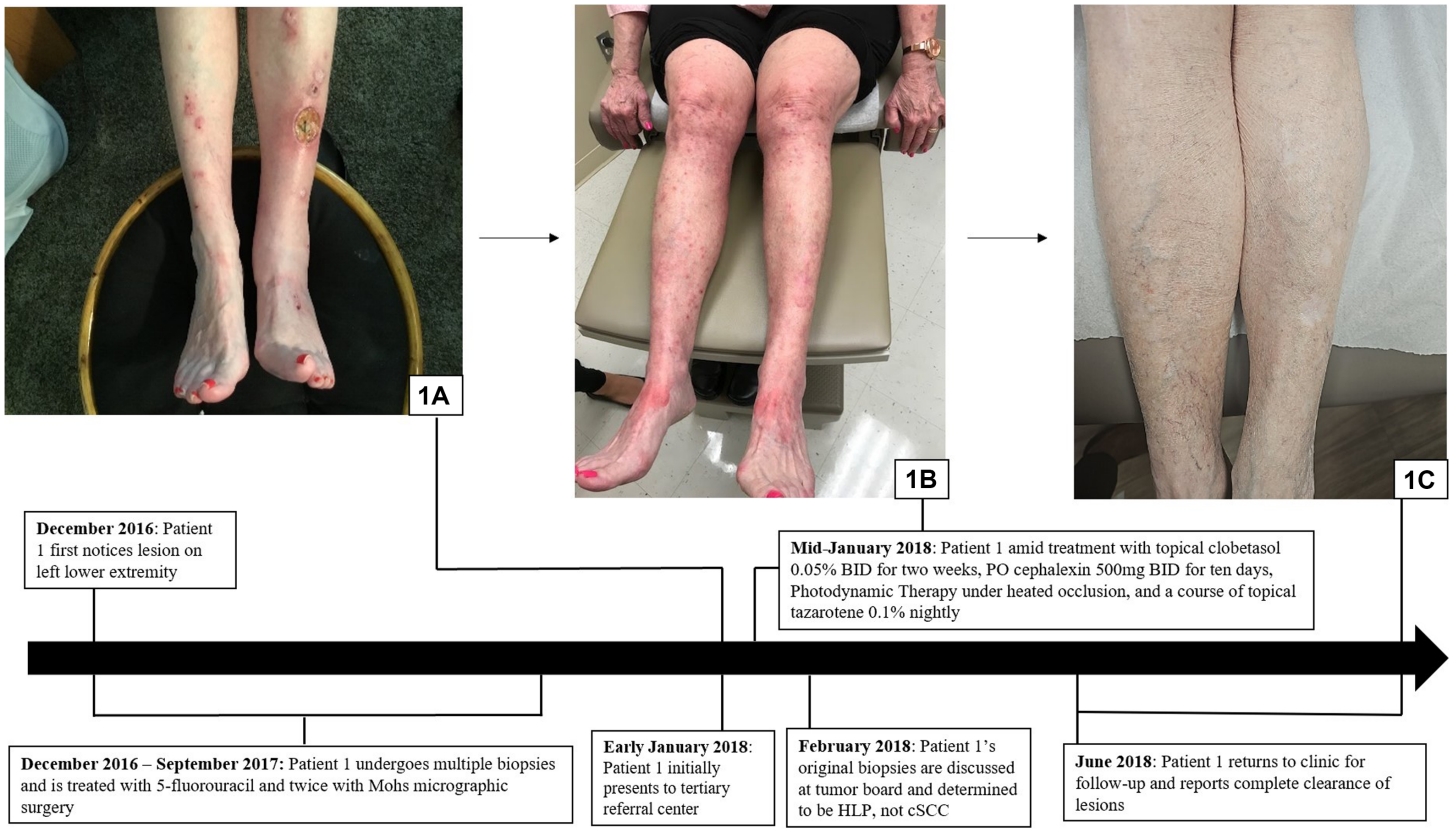
Hypertrophic lichen planus: **(A)** Initial presentation with scaly pink papules and plaques on the exam in patient 1. **(B)** Patient 1 in the midst of treatment with clobetasol, cephalexin, PDT under heated occlusion, and a course of tazarotene, resulting in, **(C)** complete clearance.

A 72-year-old woman with a past medical history of essential thrombocytopenia presented with a single hyperkeratotic papule on her left lower leg. Biopsy revealed cSCC and she was treated with Mohs micrographic surgery and 5-fluorouracil. Subsequently, the patient developed three new hyperkeratotic lesions that were also treated with Mohs micrographic surgery after biopsies revealed cSCC. The patient noted that these surgeries caused her excessive fatigue and impacted her ability to carry out her day-to-day activities. Soon after, the patient noticed the development of new lesions on her bilateral upper and lower extremities, back, and upper chest (Figure 1A). The patient was referred to a tertiary referral center due to the challenge of treating her “multifocal cSCC,” where a review at the Cutaneous Oncology Tumor Board of the original three biopsies revealed findings consistent with HLP.

The patient was diagnosed with HLP and was initiated for treatment with clobetasol 0.05% ointment twice daily for 2 weeks, cephalexin of 500 mg orally twice daily for 10 days, photodynamic therapy (PDT) under heated occlusion, and a 6-week course of tazarotene 0.1% cream, resulting in complete resolution of all lesions (Figures 1B,C).

### Patient 2

**Figure 2:**
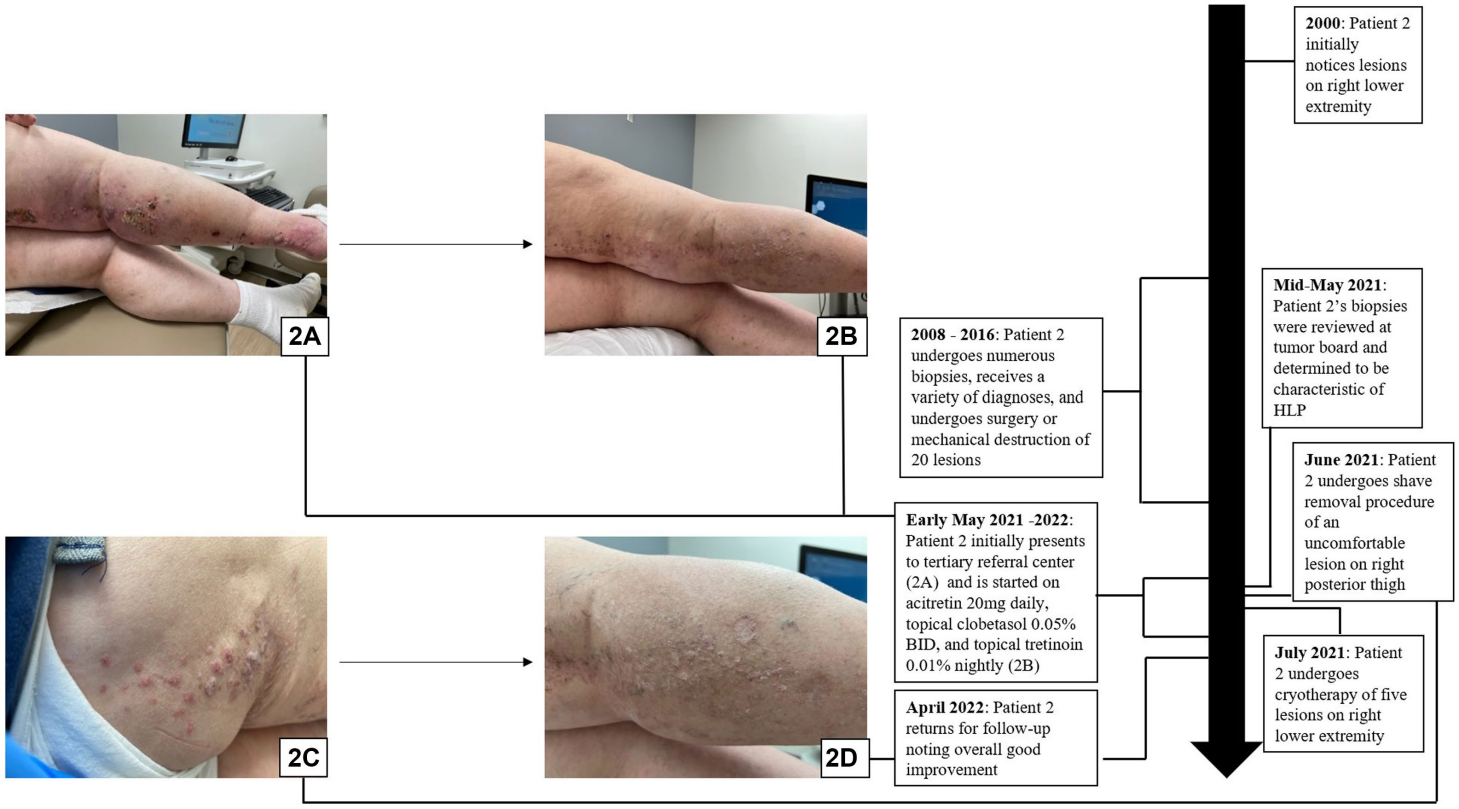
Hypertrophic lichen planus: **(A,B)** Initial presentation with scaly pink papules and plaques on exam in patient 2. **(C,D)** Patient 2 in the midst of treatment with acitretin, clobetasol, tretinoin, shave removal, and cryotherapy with overall good improvement on follow-up.

A 60-year-old woman with a past medical history significant for basal cell carcinoma (BCC) and small bowel and endometrial adenocarcinomas presented to the clinic with a 12 cm hyperpigmented and erythematous plaque studded with hyperkeratotic papulonodules that extended from the right inguinal fold to the right anterior thigh in a blaschkoid distribution. Her right posterior leg also had a large linear plaque studded with hyperkeratotic papules (Figures 2A,B). Further discussion with the patient revealed that many biopsies had been performed over an 8-year period from 2008 to 2016, leading to an array of diagnoses including keratoacanthoma (KA), actinic keratosis, invasive SCC, clear cell carcinoma, and sebaceous carcinoma. The patient noted that her lower extremity surgeries had a protracted course of healing, and each surgery was painful, impacting her comfort. The case and associated biopsies were reviewed at the Cutaneous Oncology Tumor Board, and the patient was diagnosed with linear HLP.

It is important to note that despite the patient’s medical history of BCC and adenocarcinomas, genetic studies did not reveal *mutL homolog 1* or *mutS homolog 2* mutations, and family history was not concerning for Lynch Syndrome.

The patient was treated with acitretin 20 mg daily, clobetasol 0.05% cream every morning, and tretinoin 0.1% cream every night, along with shave removal of a 2.3 cm lesion on the right posterior thigh and cryotherapy of several hyperkeratotic lesions (Figure 2C). The patient returned for a follow-up appointment 6 months later, exhibiting overall good improvement (Figure 2D).

### Patient 3

A 68-year-old woman presented with two 1 cm pink lesions on the right shin and right anterior thigh. The lesion on the right shin was biopsied, revealing KA. The patient was treated with Mohs micrographic surgery with secondary intention wound management and a prolonged healing course, limiting her ability to maintain her swimming regimen.

Approximately 6 months after the initial presentation, the patient noted scattered pink scaly papules and plaques on her neck, back, and bilateral upper extremities, with a cluster of lesions on the right lower extremity around the previous Mohs site. Biopsy of these lesions on the right shin and the right anterior thigh once again showed KA. Due to the clinical picture of multifocal inflammatory lesions, the biopsies underwent internal review at the Cutaneous Oncology Tumor Board, where they were determined to be HLP.

The patient was treated with acitretin 10 mg three times weekly, and the frequency was increased to 10 mg daily 3 months later. In subsequent follow-up appointments, the patient reported complete clearance of the lesions. The patient chose to self-discontinue acitretin 7 months after the diagnosis of HLP and is currently following up with her primary dermatologist.

## Discussion

A PubMed search of articles indexed for MEDLINE and a Google Scholar search using the terms “hypertrophic lichen planus” and “squamous cell carcinoma” revealed fewer than 26 reported cases of HLP misdiagnosed as cSCC (Supplementary Table S1, which lists literature describing lichen planus misdiagnosed as squamous cell carcinoma). HLP and cSCC share clinical and histopathological features, which can lead to the misdiagnosis of HLP as cSCC (12–15). This diagnostic dilemma is further complicated by evidence that HLP can undergo malignant transformation and focally progress to cSCC (6, 8, 9). Our case series demonstrates the importance of assessing the overall clinical picture in the setting of presumed multifocal cSCC to avoid non-indicated procedures.

In our case series, initial misdiagnosis for each patient was likely due to a combination of clinical and histopathological similarities between HLP and cSCC, and a lack of clinicopathological correlation. The mean duration of time with an SCC diagnosis prior to an HLP diagnosis was 4 years (range 1–8 years). After correctly identifying HLP in our patients, therapy was initiated with oral or topical retinoids and high-potency topical steroids, with a mean improvement time of 6 weeks (Table 1).

**Table 1:** Summary of key clinical features, diagnostic challenges, and treatment outcomes for patients 1, 2, and 3.

	Clinical features	Diagnostic challenges	Treatment outcomes
Patient 1	Hyperkeratotic lesions on bilateral upper and lower extremities, back, and upper chest.	Multiple rounds of Mohs surgery after biopsies were misdiagnosed with cSCC caused fatigue and impacted the patient’s ability to carry out day-to-day activities.	Treated with clobetasol 0.05% ointment twice daily for 2 weeks, cephalexin 500 mg orally twice daily for 10 days, PDT under heated occlusion, and a 6 week course of tazarotene 0.1% cream, resulting in complete resolution of all lesions.
Patient 2	A 12 cm hyperpigmented, erythematous plaque studded with hyperkeratotic papulonodules that extended from the right inguinal fold to the right anterior thigh in a blaschkoid distribution. The right posterior leg had a large linear plaque studded with hyperkeratotic papules.	Many biopsies were performed over 8 years, leading to an array of diagnoses. Lower extremity surgeries had a protracted course of healing, and each surgery was painful, impacting the patient’s comfort.	Treated with acitretin 20 mg daily, clobetasol 0.05% cream every morning, and tretinoin 0.1% cream every night, along with shave removal of a 2.3 cm lesion on the right posterior thigh and cryotherapy of several hyperkeratotic lesions. The patient noted overall good improvement on follow-up 6 months later.
Patient 3	Pink scaly papules and plaques on the neck, back, and bilateral upper extremities, with a cluster of lesions on the right lower extremity around the previous Mohs site.	The patient was previously treated with Mohs micrographic surgery with secondary intention wound management, with a prolonged healing course, limiting her ability to maintain her swimming regimen.	Treated with acitretin 10 mg three times weekly, and the frequency was increased to 10 mg daily 3 months later. The patient reported complete clearance of the lesions in follow-up appointments. The patient chose to self-discontinue acitretin 7 months after the diagnosis of HLP and is currently following up with her primary dermatologist.

**Figure 3:**
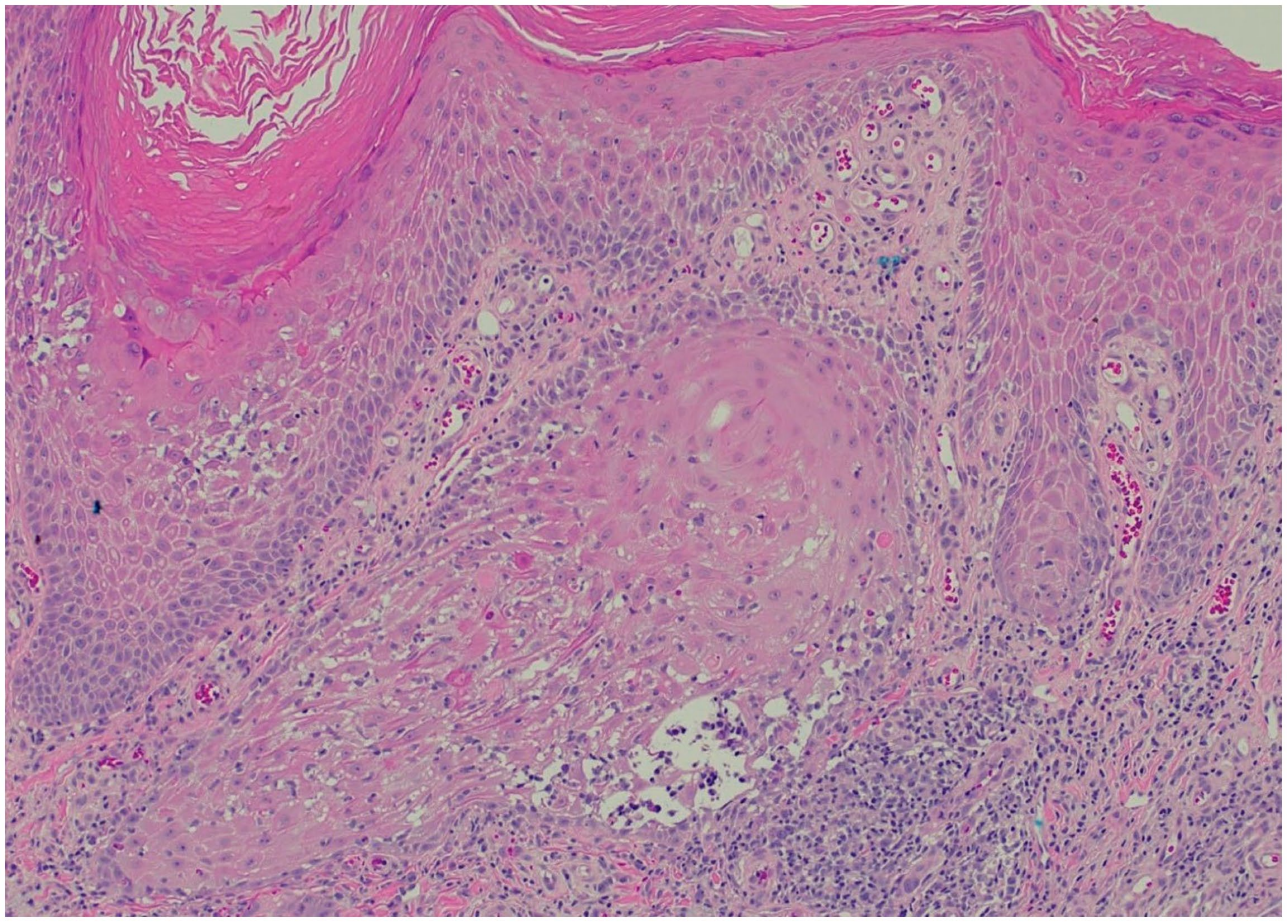
Representative histopathological image for our patients (H&E stain): saw-toothed rete ridges and a band-like lymphohistiocytic infiltrate at the dermoepidermal junction, along with wedge-shaped hypergranulosis and civatte bodies.

Clinically, HLP plaques are frequently located on the distal lower extremities and are associated with follicular accentuation (4, 5). Additionally, patients may have no history of sun damage and no predisposing risk factors for cSCC (13). Histologically, HLP is notable for hyperorthokeratosis, wedge-shaped hypergranulosis, and irregular psoriasiform hyperplasia limited to the epidermis (Figure 3), while cSCC presents with cytologic atypia, extension beyond the superficial dermis, and other invasive characteristics (6, 13).

The pathogenesis of HLP is T-cell-mediated, involving the Janus kinase 2 (JAK2)/the signal transducer and activator of transcription 1 (STAT1) pathways. IFN-γ, IL-21, and pSTAT1 have been found in the dermal infiltrate of HLP lesions (16, 17). IFN-γ was shown to prime keratinocytes and increase keratinocyte susceptibility to CD8^+^ T-cell-mediated cytotoxicity through the induction of major histocompatibility complex expression. These cell-mediated cytotoxic responses are dependent on JAK2 and STAT1 signaling, as demonstrated by JAK2 and STAT1 knockouts, which protected IFN-γ-primed keratinocytes from cell-mediated cytotoxic responses (17). These recent developments have supported the use of topical and systemic JAK inhibitors in cutaneous inflammatory and immune-mediated processes (18). Furthermore, other recently published studies have identified *SPRR1B*, *miRNA27b*, and *miRNA137* gene expressions and a − 308 G/A polymorphism in TNFα as potential biomarkers for oral lichen planus (19, 20). Additional future directions for HLP pathogenesis research include the identification of potential genetic biomarkers to further differentiate HLP from cSCC.

The current first-line therapy for HLP is topical corticosteroids; however, second-line non-steroidal treatments, such as acitretin, sulfasalazine, griseofulvin, topical calcineurin inhibitors, and narrow-band ultraviolet B radiation, are also associated with increased response rates (5, 21). With advancements in understanding HLP pathogenesis, JAK inhibitors, such as tofacitinib and baricitinib, have been successfully adopted into some lichen planus treatment plans and have served as effective therapeutic agents (17, 22, 23). Additionally, the IL-4Rα antagonist and biological immunomodulator dupilumab shows promise as an effective therapy for lichen planus (24–27). JAK inhibitors and dupilumab should be further evaluated in randomized-controlled trials for widespread use in the treatment of HLP. Treatments for cSCC differ from those of HLP and include, but are not limited to, cryotherapy, laser therapy, excision, and Mohs micrographic surgery, depending on the size, location, and aggressiveness of the lesion (23).

## Conclusion

HLP is a chronic cutaneous subtype of lichen planus, a T-cell-mediated autoimmune disorder. Clinically, HLP presents as pruritic, hypertrophic papules and plaques on the lower limbs and can significantly impair patient quality of life. Patients with HLP share clinical and histopathological findings with cSCC, including pseudoepitheliomatous hyperplasia, often leading to misdiagnosis. Misdiagnosis can lead to inadequate treatment strategies and further affect patient wellbeing. The current effective first-line treatment for HLP is topical corticosteroids. Recent research into the pathogenesis of HLP implicates the JAK2/STAT1 pathway in increasing keratinocyte susceptibility to CD8^+^ T-cell-mediated cytotoxic responses. These advancements suggest JAK inhibitors may be effective in treating HLP, and in fact, there have been recent case reports of successful HLP treatment with JAK inhibitors. Additional future directions may include the identification of potential biomarkers of HLP, which may guide therapy. Finally, a comprehensive review of the patient’s presentation is vital to identifying features consistent with a diagnosis of HLP, as misdiagnosis as cSCC may result in unnecessary interventions that can increase patient morbidity and the use of medical resources.

## Data availability statement

The original contributions presented in the study are included in the article/Supplementary material, further inquiries can be directed to the corresponding author/s.

## Ethics statement

Written informed consent was obtained from the individual(s) for the publication of any potentially identifiable images or data included in this article.

## Author contributions

NK: Writing – original draft, Writing – review & editing. LL: Supervision, Writing – original draft, Writing – review & editing. PS: Writing – original draft. JD: Conceptualization, Supervision, Writing – original draft, Writing – review & editing.

## References

[1] Eisen D. The evaluation of cutaneous, genital, scalp, nail, esophageal, and ocular involvement in patients with oral lichen planus. Oral Surg Oral Med Oral Pathol Oral Radiol Endod. (1999) 88:431–6. doi: 10.1016/s1079-2104(99)70057-0, PMID: 10519750

[2] Ioannides D Vakirlis E Kemeny L Marinovic B Massone C Murphy R. European S1 guidelines on the management of lichen planus: a cooperation of the European dermatology forum with the European academy of dermatology and venereology. J Eur Acad Dermatol Venereol. (2020) 34:1403–14. doi: 10.1111/jdv.16464 32678513

[3] Le Cleach L Chosidow O. Clinical practice. Lichen planus. N Engl J Med. (2012) 366:723–32. doi: 10.1056/NEJMcp1103641 22356325

[4] Gorouhi F Davari P Fazel N. Cutaneous and mucosal lichen planus: a comprehensive review of clinical subtypes, risk factors, diagnosis, and prognosis. Sci World J. (2014) 2014:742826:1–22. doi: 10.1155/2014/742826 PMC3929580 24672362

[5] Solimani F Forchhammer S Schloegl A Ghoreschi K Meier K. Lichen planus - a clinical guide. J Dtsch Dermatol Ges. (2021) 19:864–82. doi: 10.1111/ddg.14565, PMID: 34096678

[6] Haenen CCP Buurma AAJ Genders RE Quint KD. Squamous cell carcinoma arising in hypertrophic lichen planus. BMJ Case Rep. (2018) 2018:bcr 2017224044. doi: 10.1136/bcr-2017-224044, PMID: 30002207 PMC6047711

[7] Zayour M Lazova R. Pseudoepitheliomatous hyperplasia: a review. Am J Dermatopathol. (2011) 33:112–22; quiz 123-6. doi: 10.1097/DAD.0b013e3181fcfb47 21399447

[8] Kutlubay Z Kocaturk E Demirkesen C Kavala M Sarigul S Zindanci I. Squamous cell carcinoma arising from hypertrophic lichen planus. Eur J Dermatol. (2009) 19:175–6. doi: 10.1684/ejd.2008.0590, PMID: 19106049

[9] Knackstedt TJ Collins LK Li Z Yan S Samie FH. Squamous cell carcinoma arising in hypertrophic lichen planus: a review and analysis of 38 cases. Dermatologic Surg. (2015) 41:1411–8. doi: 10.1097/DSS.0000000000000565 26551772

[10] Van Cranenburgh OD Nijland SB De Korte J Lindeboom R De Rie MA Ter Stege JA. Satisfaction with treatment and health-related quality of life among patients with lichen planus: a web-based survey. Eur J Dermatol. (2016) 26:113–6. doi: 10.1684/ejd.2015.2703, PMID: 26771827

[11] Motamed-Sanaye A Khazaee YF Shokrgozar M Alishahi M Ahramiyanpour N Amani M. JAK inhibitors in lichen planus: a review of pathogenesis and treatments. J Dermatolog Treat. (2022) 33:3098–103. doi: 10.1080/09546634.2022.2116926, PMID: 35997540

[12] Dietert JB Rabkin MS Joseph AK. Squamous cell carcinoma versus hypertrophic lichen planus; a difficult differential diagnosis of great significance in approach to treatment. Dermatologic Surg. (2017) 43:297–9. doi: 10.1097/DSS.0000000000000886, PMID: 28165351

[13] Levandoski KA Nazarian RM Asgari MM. Hypertrophic lichen planus mimicking squamous cell carcinoma: the importance of clinicopathologic correlation. JAAD Case Rep. (2017) 3:151–4. doi: 10.1016/j.jdcr.2017.01.020, PMID: 28374001 PMC5367790

[14] Astudillo MG Hoang MP Nazarian RM Foreman RK. Distinction between hypertrophic lichen planus and squamous cell carcinoma requires clinicopathologic correlation in difficult cases. Am J Dermatopathol. (2021) 43:349–55. doi: 10.1097/DAD.0000000000001776 33395040

[15] Tan E Malik R Quirk CJ. Hypertrophic lichen planus mimicking squamous cell carcinoma. Australas J Dermatol. (1998) 39:45–7. doi: 10.1111/j.1440-0960.1998.tb01242.x, PMID: 9529690

[16] Pietschke K Holstein J Meier K Schäfer I Müller-Hermelink E Gonzalez-Menendez I. The inflammation in cutaneous lichen planus is dominated by IFN-ϒ and IL-21-a basis for therapeutic JAK1 inhibition. Exp Dermatol. (2021) 30:262–70. doi: 10.1111/exd.14226, PMID: 33113249

[17] Shao S Tsoi LC Sarkar MK Xing X Xue K Uppala R. IFN-γ enhances cell-mediated cytotoxicity against keratinocytes via JAK2/STAT1 in lichen planus. Sci Transl Med. (2019) 11:eaav7561. doi: 10.1126/scitranslmed.aav7561, PMID: 31554739 PMC7285657

[18] Solimani F Meier K Ghoreschi K. Emerging topical and systemic JAK inhibitors in dermatology. Front Immunol. (2019) 10:2847. doi: 10.3389/fimmu.2019.02847, PMID: 31849996 PMC6901833

[19] Aghbari SMH Gaafar SM Shaker OG Ashiry SE Zayed SO. Evaluating the accuracy of microRNA27b and microRNA137 as biomarkers of activity and potential malignant transformation in oral lichen planus patients. Arch Dermatol Res. (2018) 310:209–20. doi: 10.1007/s00403-018-1805-0, PMID: 29368136

[20] Geng L Zhang X Tang Y Gu W. Identification of potential key biomarkers and immune infiltration in Oral lichen planus. Dis Markers. (2022) 2022:7386895–20. doi: 10.1155/2022/7386895, PMID: 35256894 PMC8898126

[21] Atzmony L Reiter O Hodak E Gdalevich M Mimouni D. Treatments for cutaneous lichen planus: a systematic review and meta-analysis. Am J Clin Dermatol. (2016) 17:11–22. doi: 10.1007/s40257-015-0160-6 26507510

[22] Seiringer P Lauffer F Pilz AC Boehmer D Biedermann T Eyerich K. Tofacitinib in hypertrophic lichen planus. Acta Derm Venereol. (2020) 100:adv00220. doi: 10.2340/00015555-3585, PMID: 32618349 PMC9201967

[23] Work Group; Invited Reviewers Kim JYS Kozlow JH Mittal B Moyer J Olenecki T Rodgers P. Guidelines of care for the management of cutaneous squamous cell carcinoma. J Am Acad Dermatol. (2018) 78:560–78. doi: 10.1016/j.jaad.2017.10.007, PMID: 29331386 PMC6652228

[24] Pousti BT Jin A Sklovar L Savage KT Zhai LL Mollanazar NK. Dupilumab for the treatment of lichen planus. Cutis. (2021) 107:E8–E10. doi: 10.12788/cutis.0232, PMID: 34096853

[25] Ch'en PY Song EJ. Lichen planus pemphigoides successfully treated with dupilumab. JAAD Case Rep. (2022) 31:56–8. doi: 10.1016/j.jdcr.2022.11.011, PMID: 36505033 PMC9732113

[26] Kazemi S Murphrey M Hawkes JE. Rapid resolution of widespread cutaneous lichen planus and generalized pruritus in an elderly patient following treatment with dupilumab. JAAD Case Rep. (2022) 30:108–10. doi: 10.1016/j.jdcr.2022.10.019, PMID: 36439781 PMC9681636

[27] Gajraj FA Zahir J Adereti C Gajraj MH. A case report and literature review of the role of dupilumab in the Management of Lichen Planus: cause or treatment? Cureus. (2023) 15:e41274. doi: 10.7759/cureus.41274, PMID: 37533618 PMC10392291

