# Supplementary material for: Case report: Hypertrophic lichen planus initially misdiagnosed as squamous cell carcinoma
Source: Front Med (Lausanne). 2024 May 15;11:1342501. doi: 10.3389/fmed.2024.1342501 (PMC11133675; doi:10.3389/fmed.2024.1342501)
Supplement: Supplementary file 1 [file Table_1.docx]

SUPPLEMENTAL TABLE 1

| **Cases of HLP misdiagnosed as SCC** | **Number of Cases** |
| --- | --- |
| Our Cases | 3 cases |
| Ameri AH, Foreman RK, Vedak P, Chen S, Miller DM, Demehri S. Hypertrophic Lichen Planus with Histological Features of Squamous Cell Carcinoma Associated with Immune Checkpoint Blockade Therapy. Oncologist. 2020 May;25(5):366-368. doi: 10.1634/theoncologist.2019-0796. Epub 2020 Feb 19. PMID: 32073194; PMCID: PMC7216465. | 2 cases |
| Amin, Mina, and Farah Abdulla. Hypertrophic lichen planus misinterpreted as squamous cell carcinoma. Skin Cancer Symposium, 2021. | 1 case |
| Astudillo MG, Hoang MP, Nazarian RM, Foreman RK. Distinction Between Hypertrophic Lichen Planus and Squamous Cell Carcinoma Requires Clinicopathologic Correlation in Difficult Cases. Am J Dermatopathol. 2021 May 1;43(5):349-355. doi: 10.1097/DAD.0000000000001776. PMID: 33395040. | 1 case |
| Coscarart A, Martel J, Lee MP, Wang AR. Pembrolizumab-induced pseudoepitheliomatous eruption consistent with hypertrophic lichen planus. J Cutan Pathol. 2020 Mar;47(3):275-279. doi: 10.1111/cup.13587. Epub 2019 Oct 20. PMID: 31589773. | 1 case |
| Dietert JB, Rabkin MS, Joseph AK. Squamous Cell Carcinoma Versus Hypertrophic Lichen Planus; a Difficult Differential Diagnosis of Great Significance in Approach to Treatment. Dermatol Surg. 2017 Feb;43(2):297-299. doi: 10.1097/DSS.0000000000000886. PMID: 28165351. | 1 case |
| Fontecilla NM, Kittler NW, Lopez A, Yang C, Geskin L. Programmed cell death protein-1 inhibitor-induced granuloma annulare and hypertrophic lichen planus masquerading as squamous cell carcinoma. JAAD Case Rep. 2018 Jul 24;4(7):636-639. doi: 10.1016/j.jdcr.2018.01.020. PMID: 30094305; PMCID: PMC6073079. | 1 case |
| Haslam, S. P., Ross, L. S., Lowe, A. C., Kelly, B. C. (2017). Hypertrophic Lichen Planus versus Well-Differentiated Squamous Cell Carcinoma: A Histological Challenge . *SKIN The Journal of Cutaneous Medicine*, *1*(2), 91–94. https://doi.org/10.25251/skin.1.2.6. | 2 cases |
| Levandoski KA, Nazarian RM, Asgari MM. Hypertrophic lichen planus mimicking squamous cell carcinoma: The importance of clinicopathologic correlation. JAAD Case Rep. 2017 Mar 21;3(2):151-154. doi: 10.1016/j.jdcr.2017.01.020. PMID: 28374001; PMCID: PMC5367790. | 1 case |
| Mozafari N, Bidari-Zerehpoosh F, Movahedi M, Dadkhahfar S. Hypertrophic lichen planus on lip mimicking SCC. Clin Case Rep. 2022 Aug 8;10(8):e6191. doi: 10.1002/ccr3.6191. PMID: 35957792; PMCID: PMC9360339. | 1 case |
| Murad W, Fekrazad MH. Hypertrophic lichen planus mimicking squamous cell carcinoma of skin. J Am Osteopath Assoc. 2014 Sep;114(9):735. doi: 10.7556/jaoa.2014.144. PMID: 25170045. | 1 case |
| Myrdal CN, Sundararajan S, Curiel-Lewandrowski C. Widespread Hypertrophic Lichen Planus following Programmed Cell Death Ligand 1 Blockade. Case Rep Dermatol. 2020 Jun 18;12(2):119-123. doi: 10.1159/000508490. PMID: 32774246; PMCID: PMC7383211. | 1 case |
| Nguyen R, Murray B, McCormack C. Hypertrophic lichen planus: masquerading as squamous cell carcinoma. Clinical Dermatology. 2014;2(3):136-9. | 2 cases |
| Shao EX, Carew B, Muir J. Hypertrophic lichen planus mistaken for squamous cell carcinoma. Med J Aust. 2018 Nov 19;209(10):462. doi: 10.5694/mja18.00577. PMID: 30428823. | 3 Cases |
| Smirnov B, Bowles AA, Strasswimmer JM, Nousari CH. Lupus Erythematosus Lichen Planus Overlap Syndrome Mimicking Squamous Cell Carcinoma. J Clin Aesthet Dermatol. 2019 Sep;12(9):36-38. Epub 2019 Sep 1. PMID: 31641416; PMCID: PMC6777702. | 1 case |
| Tan E, Malik R, Quirk CJ. Hypertrophic lichen planus mimicking squamous cell carcinoma. Australas J Dermatol. 1998 Feb;39(1):45-7. doi: 10.1111/j.1440-0960.1998.tb01242.x. PMID: 9529690. | 1 case |
| Totonchy MB, Leventhal JS, Ko CJ, Leffell DJ. Hypertrophic Lichen Planus and Well-Differentiated Squamous Cell Carcinoma: A Diagnostic Conundrum. Dermatol Surg. 2018 Nov;44(11):1466-1470. doi: 10.1097/DSS.0000000000001465. PMID: 29360655. | 3 cases |

**Supplemental Table 1.** List of previously published literature describing cases of lichen planus misdiagnosed as squamous cell carcinoma.
